# Successful Tocilizumab Therapy for Macrophage Activation Syndrome Associated with Adult-Onset Still's Disease: A Case-Based Review

**DOI:** 10.1155/2016/5656320

**Published:** 2016-09-05

**Authors:** Eri Watanabe, Hitoshi Sugawara, Takeshi Yamashita, Akira Ishii, Aya Oda, Chihiro Terai

**Affiliations:** ^1^Division of General Medicine, Department of Comprehensive Medicine 1, Saitama Medical Center, Jichi Medical University, 1-847 Amanuma-cho, Omiya-ku, Saitama 330-8503, Japan; ^2^Division of Rheumatology, Department of Comprehensive Medicine 1, Saitama Medical Center, Jichi Medical University, 1-847 Amanuma-cho, Omiya-ku, Saitama 330-8503, Japan; ^3^Division of Rheumatology, IMSUT Hospital, The Institute of Medical Science, The University of Tokyo, 4-6-1 Shirokanedai, Minato-ku, Tokyo 108-8639, Japan

## Abstract

We report the case of a 71-year-old Japanese woman with adult-onset Still's disease (AOSD) in whom macrophage activation syndrome (MAS) developed despite therapy with oral high-dose prednisolone and intravenous methylprednisolone pulse therapy twice. She was successfully treated with tocilizumab (TCZ). Soon afterward, her fever ceased and high levels of both ferritin and C-reactive protein levels decreased. Her course was complicated by disseminated intravascular coagulation, cytomegalovirus infection, and* Pneumocystis jirovecii* pneumonia. After these were resolved, AOSD-associated MAS was well controlled. She was discharged on hospital day 87. Although biologics such as TCZ are becoming established for the treatment of AOSD, there is no recommended therapy for AOSD-associated MAS. Several biologics have been tried for this complication, but their efficacy and safety remain controversial. We reviewed reported cases of AOSD-associated MAS successfully treated with various biologics. TCZ initiation after adequate nonselective immunosuppressive therapy, such as methylprednisolone pulse therapy or a prednisolone-based combination of immunosuppressants, can be an effective treatment for AOSD-associated MAS. On the other hand, biologics given after insufficient immunosuppressive therapy may cause MAS. A strategy combining adequate immunosuppression and a biologic could be safe if special attention is given to adverse events such as opportunistic infections or biologic-associated MAS.

## 1. Introduction

Adult-onset Still's disease (AOSD) is a rare systemic febrile inflammatory disorder of unknown etiology, with an estimated prevalence of 0.16 cases per 100,000 in western France [1], 0.4 cases per 100,000 in northern Norway [2], and 3.7 cases per 100,000 in Japan [3]. AOSD is characterized by four major symptoms: high spiking fever, arthralgia or arthritis, evanescent salmon-pink maculopapular rash, and hyperleukocytosis [4]. It is essentially diagnosed by exclusion of a wide range of disorders, including infection, malignancy, and autoimmune conditions [5].

AOSD can cause macrophage activation syndrome (MAS) [3], which is an acute systemic inflammatory syndrome characterized by cytopenia, organ dysfunction, and coagulopathy associated with an excessive activation of macrophages, but there are no classification criteria of AOSD-associated MAS. AOSD is believed to closely resemble systemic juvenile idiopathic arthritis (sJIA) [5]. The 2016 classification criteria for MAS complicating sJIA [6] include hyperferritinemia (>684 ng/mL) and any two of the following: thrombocytopenia (≤181 × 10^9^/liter), a high aspartate aminotransferase (AST) level (>48 units/liter), hypertriglyceridemia (>156 mg/dL), and a low fibrinogen level (≤360 mg/dL), regardless of the presence or absence of pathologically detected hemophagocytosis. The prevalence of MAS in AOSD is 7.7%–16% [3, 7, 8]. Possible triggering factors of MAS are reported to be the primary disease activity (75%), infection (18.8%), and drugs such as antibiotics (6.3%) [9].

MAS and hemophagocytic syndrome (HPS) are two closely-related clinicopathological entities that present as potentially life-threatening complications of AOSD. MAS is the preferred term for reactive or secondary HPS in underlying rheumatologic disorders [10]. Bone marrow hemophagocytosis took up no more than 47.8% of the patients with AOSD-associated MAS in 94 cases by literature review [10].

Disseminated intravascular coagulation (DIC) is a serious complication of both AOSD and AOSD-associated MAS and is associated with severe liver cytolysis and high mortality [11]; the mortality rate of AOSD-associated MAS is ranging between 9.5% [9] and 22% [5].

The utility of cytokine-targeted biologic agents in the treatment of AOSD-associated MAS remains unclear [12]. The use of TCZ for treatment of refractory AOSD is recommended by Asanuma et al. [3] and Elkayam et al. [13]. But there is no established standard therapy for AOSD-associated MAS, and several treatments have been tried, for example, biologics targeting tumor necrosis factor-*α* (TNF-*α*) [etanercept (ETN), infliximab (IFX), and adalimumab], IL-1 [anakinra (ANK) and canakinumab], and IL-6 [tocilizumab (TCZ)] [14–16]. Recently, Lenert and Yao reported early addition of ANK, systemic glucocorticoids, and cyclosporine as a triple regimen might improve clinical outcomes of AOSD-associated MAS [10]. Other reports describe MAS was aggravated during biologic treatments for refractory AOSD [3, 12, 17–20]. It follows that the efficacy and safety of biologics used for this condition still remain controversial.

We report the case of a patient with AOSD-associated MAS who had undergone nonselective immunosuppressive therapy with high-dose oral prednisolone (PSL) and intravenous methylprednisolone (mPSL) pulse therapy twice but who only improved after TCZ was administered. For improving clinical outcomes by prompt management, we review previously reported cases of AOSD-associated MAS in which the pathological evidence of hemophagocytosis was documented and treatment with a biologic succeeded.

## 2. Case Report

A 71-year-old Japanese woman was admitted to our medical center with fever, general fatigue, and skin rash. Four weeks before admission, she had spiking fever, sore throat, general fatigue, and loss of appetite, all of which gradually worsened. She did not take any medication for the symptoms, and she had no significant family history. Three weeks before admission, she developed a skin rash concurrent with the fever and was kept under intensive observation at two different hospitals during those 3 weeks. Laboratory data from the previous two hospitals included a clear urinalysis and elevated values for the white blood cell (WBC) count (12,000/*μ*L), lactate dehydrogenase (LDH, 2,114 units/liter), AST (188 units/liter), alanine aminotransferase (ALT, 84 units/liter), C-reactive protein (CRP, 12.97 mg/dL), and serum ferritin (80,752 ng/mL). Anti-Epstein–Barr virus, anti-cytomegalovirus (CMV), and anti-human parvovirus B19 IgG levels suggested latent exposures. Tests for rheumatoid factor (RF) and antinuclear antibodies (ANAs), including other autoantibodies, were negative. Chest X-ray, abdominal ultrasound, echocardiography, and whole-body contrast-enhanced computed tomography (CT) revealed neither obvious infections nor tumors. Microbiology cultures of blood, urine, cerebrospinal fluid, and bone marrow were negative for infective bacteria and fungi. Polymerase chain reaction assays for both tuberculosis and atypical mycobacteria in the gastric fluid were negative. A random skin biopsy for detecting intravascular lymphoma was negative as well. No specific diagnosis was made, and she was referred to our hospital for the evaluation of fever of unknown origin.

On admission, she had fatigue, fever, sore throat, and bilateral shoulder pain. Her temperature was 38.7°C. The submandibular lymph nodes were bilaterally enlarged. An evanescent salmon-pink erythema was observed around the neck, back, and lower extremities only when she had fever.

The laboratory data were as follows: WBC 11,520/*μ*L (bands, 41%; segments, 57%; lymphocytes, 1%; and monocytes, 1%), red blood cells 2,850,000/*μ*L, hemoglobin 8.7 g/dL, platelets 78 × 10^9^/liter, total protein 4.5 g/dL, albumin 1.6 g/dL, AST 63 units/liter, ALT 32 units/liter, LDH 1,178 units/liter, CRP 15.2 mg/dL, ferritin 58,740 ng/mL, ANA titer 1 : 40, RF ≤ 11 IU/mL, prothrombin time international normalization ratio (INR) 0.95, and activated partial thromboplastin time (APTT) 30.3 s. A CT scan showed bilateral pleural effusions and mild splenomegaly. Blood and urine cultures were negative.

The findings of spiking fever, arthralgia, evanescent rash, leukocytosis, sore throat, lymphadenopathy, elevated serum transaminases, and negative results for ANAs and RF fulfilled four major and four minor criteria for the diagnosis of AOSD proposed by Yamaguchi et al. [4].

**Figure 1:**
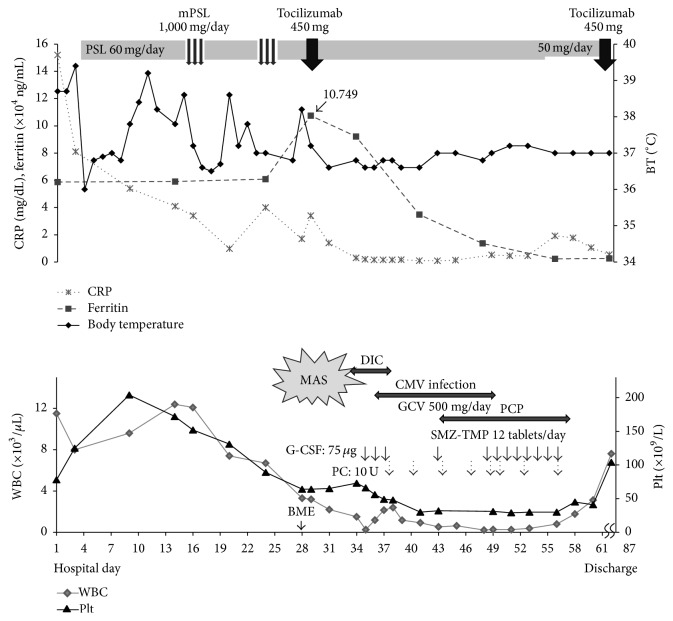
Clinical course during hospitalization. CRP, C-reactive protein; WBC, white blood cell; Plt, platelet; PSL, prednisolone; mPSL, methylprednisolone; PIPC/TAZ, piperacillin/tazobactam; CFPM, cefepime; MAS, macrophage activation syndrome; DIC, disseminated intravascular coagulation; CMV, cytomegalovirus; GCV, ganciclovir; PCP,* Pneumocystis jirovecii* pneumonia; SMZ-TMP, sulfamethoxazole/trimethoprim; G-CSF, granulocyte colony-stimulating factor; PC, platelet concentrate; BME, bone marrow examination.

The patient's course in our hospital is shown in Figure 1. On hospital day (HD) 4, a daily dose of 60 mg [1 mg/kg body weight (BW)] of oral PSL was started. The fever fell down to normal level temporarily, but a high spiking fever and leukocytosis recurred on HD 11. She was treated with mPSL pulse therapy (1,000 mg/day) intravenously for 3 days starting on HD 16, followed by 60 mg/day of oral PSL. The fever ceased immediately and WBC count decreased. However, the high spiking fever recurred on HD 20, and CRP levels rebounded on HD 24. A second course of mPSL pulse therapy was given, resulting in improvement of the fever and reduction of CRP levels.

On HD 28, hyperferritinemia (107,490 ng/mL), pancytopenia (WBC, 2,430/*μ*L; hemoglobin, 7.4 g/dL; and platelets, 68 × 10^9^/liter), and elevations of AST (212 units/liter) and triglyceride (236 mg/dL) were observed, along with a high fever. These data met 2016 classification criteria for MAS complicating sJIA [6]. A bone marrow examination revealed 7.4% hemophagocytic macrophages, indicating that the patient had developed AOSD-associated MAS. After obtaining the patient's informed consent, we administered one dose of TCZ (450 mg; 8 mg/kg BW) intravenously on HD 29 in addition to oral PSL because serum IL-6 levels were elevated at 36 pg/mL (normal, ≤4.0 pg/mL). This treatment was associated with improvement in both ferritin and CRP levels, and her fever ceased.

On HD 34, the patient developed petechiae and purpura on both legs, and laboratory data showed coagulopathy (INR, ≥9.0; APTT, ≥200 s; fibrinogen ≤ 50 mg/dL; D-dimer, 5.0 *μ*g/mL; and FDP, 10.4 *μ*g/mL) and thrombocytopenia, consistent with DIC [21]. It seemed most likely that DIC was caused by CMV infection, as indicated by increased CMV-antigen positive cells (301 positive cells/slide) rather than by MAS. Ganciclovir was administered with improvements in the coagulopathy and CMV exclusion. On HD 43, she developed* Pneumocystis jirovecii* pneumonia and was treated with sulfamethoxazole/trimethoprim. She had exacerbation of her pancytopenia, primarily the leukopenia (WBC 260/*μ*L) and thrombocytopenia (66 × 10^9^/liter), which may have been because of the AOSD-associated MAS, CMV infection, or adverse effects of TCZ or the drugs administered for the CMV infection or pneumonia. The patient was treated with granulocyte colony-stimulating factor and repeated platelet transfusions until leukopenia and thrombocytopenia resolved.

Despite these life-threatening events, the administration of TCZ seemed to have resulted in reduction of the high levels of ferritin and CRP and control of her symptoms, as she no longer had spiking fevers or skin rash, indicating that the AOSD-associated MAS was well controlled. Following treatment for the concurrent problems described above, a second dose of TCZ was given uneventfully on HD 62. The patient remained in remission and was discharged on HD 87.

She had been treated on an outpatient basis with a combination of intravenous TCZ every 4 weeks and a maintenance dose of oral PSL for 8 months. After withdrawal from TCZ, she was switched to low-dose mPSL (2 mg/day) and tacrolimus (2 mg/day). At the 18-month follow-up, no sign of relapse was observed.

## 3. Discussion

This report indicates that TCZ following nonselective immunosuppressive therapy (i.e., high-dose oral PSL and intravenous mPSL pulse therapy) led to the remission of AOSD-associated MAS.

Two important clinical issues arise from the clinical course of the present patient. First, it remains to be clarified which biologic should be used for the treatment of AOSD-related MAS. Second, the safety of biologics in the treatment of AOSD-associated MAS requires further investigation.

We reviewed the literature for reports on the treatment of AOSD-associated MAS with biologics [22–27] (Table 1). We searched for literature in English and Japanese languages published up until December 2015 in PubMed database for eligible articles that simultaneously met with Medical Subject Headings terms of AOSD and MAS/HPS. We included only articles in which a biologic was used for the treatment of AOSD-associated MAS and hemophagocytosis was pathologically documented. The eight patients, including ours (number 8), included two men and six women, aged 16–71 years. The duration of AOSD ranged from 2 weeks to 2 months. Six patients responded to TCZ, five received it as the first biologic, and one (number 6) failed to respond to ANK and was switched to TCZ. In one patient (number 2), ANK was effective as the first agent. One patient (number 1) did not respond to IFX but improved when switched to ETN. Therefore, TCZ was used most frequently in successful treatment for AOSD-associated MAS. Considering the safety of biologics, only two patients (number 1 and number 8) had opportunistic infections. Patient number 6 had fulminant myocarditis, but he continued to do well on TCZ as monotherapy after recovery from a cardiac arrest. Hence, TCZ was believed to be effective [26].

Biologic-associated MAS emerges after the administration of biologics for the treatment of AOSD [17–20] (Table 2). We searched for literature in English and Japanese languages in the same way as described above. We included only articles in which a biologic was used for the treatment of AOSD, MAS was developed after biologic therapy, and hemophagocytosis was pathologically documented. There have been four reported patients with biologic-associated MAS: two were treated with ETN, one with TCZ, and one with canakinumab. None of them were treated with mPSL pulse therapy or PSL-based immunosuppression prior to the administration of biologics.

There is no consensus about the mechanisms by which biologics cause MAS. Several serum cytokines, such as TNF-*α*, IL-1*β*, IL-6, IL-18, and interferon-*γ*, are involved in AOSD and may trigger MAS [12, 14, 28–31]. IL-18 acts upstream of IL-6 in the inflammatory cytokine cascade [14, 31]. Therefore, TCZ monotherapy might be ineffective, as it is unable to fully inhibit the inflammatory cytokines downstream of IL-18 [27]. The inhibition of a single cytokine pathway may cause an unfavorable imbalance in the cytokine network involved in AOSD-associated MAS [24].

Of the patients successfully treated with biologics in Table 1, none had exacerbations of MAS while on treatment. Interestingly, for immunosuppressive therapy prior to starting a biologic, mPSL pulse therapy was given to seven patients (87.5%), and the remaining (number 5) had the combination of high-dose of PSL plus MTX. In contrast with biologics that selectively inhibit a single cytokine, mPSL pulse therapy or PSL-based combinations of immunosuppressants including MTX and CyA may inhibit multiple immune system components nonselectively. Glucocorticoids interfere with multiple cytokine pathways and suppress hypercytokinemia [32]. MTX has many immunosuppressive effects, including the inhibition of proinflammatory cytokine production, suppression of lymphocyte proliferation and neutrophil chemotaxis and adherence, and reduction of serum immunoglobulins [33, 34]. CyA acts as calcineurin inhibitor, mainly targeting helper T lymphocytes and thus inhibiting the secretion of cytokines [35]. Concomitant nonselective immunosuppressive therapy before the administration of biologics may prevent cytokine imbalance or storm in AOSD-associated MAS and contribute to the effective and safe use of biologics as was demonstrated in the cases shown in Table 1. Furthermore, these immunosuppressive strategies may also prevent the development of biologic-associated MAS.

## 4. Conclusions

TCZ after mPSL pulse therapy significantly contributed to inducing the clinical remission of AOSD-associated MAS. Before starting biologics for AOSD-associated MAS, clinicians should consider the administration of adequate nonselective immunosuppressive therapy, such as mPSL pulse therapy or PSL-based combinations of immunosuppressants, to prevent biologic agent-associated MAS. Close attention must be paid to adverse events, particularly opportunistic infections.

## Figures and Tables

**Table 1:** Case reports of biologic therapy for refractory AOSD-associated macrophage activation syndrome.

Pt	Reference	Age (years)	Sex	Disease duration of AOSD	Disease manifestations before initiation of biologics	Therapy before biologics (mg)	First biologics/response	Second biologics/response	Maintenance therapy after biologics (mg)	Adverse events after biologics
1	Maeshima et al. [22]	16	F	N.D.	F-A-R-LEU-EST-MAS	Pulse^‖^, PSL (60), etoposide, CyA (200), TAC (3), MTX (4–8)	IFX/ineffective	ETN/effective	MTX (12), PSL	PCP

2	Loh et al. [23]	20	M	2 weeks	F-R-LEU-S-L-HSM-MAS	Pulse^*‖*^, hydrocortisone	ANK/effective		CyA (100), PSL, MTX (10)	N.D.

3	Komiya et al. [24]	35	F	<2 months	F-R-LEU-S-L-HSM-EST-MAS	Pulse^‖^: 3 times, PSL (80–50), CyA (200), TAC (3), PE	TCZ/effective		PSL, TAC (3–1)	N.D.

4	de Boysson et al. [25] (2 patients)	45	F	2 weeks	F-A-R-LEU-S-HSM-MAS	Pulse^‖^, PSL (1 mg/kg), IVIG: 3 times, MTX (10)	TCZ/effective		PSL, MTX	N.D.

5	de Boysson et al. [25] (2 patients)	24	F	>2 weeks	F-A-R-LEU-S-EST-MAS	PSL (1 mg/kg), MTX (10), IVIG	TCZ/effective		PSL, MTX	N.D.

6	Savage et al. [26]	32	M	4 weeks	F-A-R-LEU-S-fulminant myocarditis-MAS	PSL (40), pulse^‖^, IVIG^a^, CyA (3 mg/kg)^a^	ANK/ineffective	TCZ/effective	N.D.	Cardiac arrest^b^ after TCZ initiation

7	Kobayashi et al. [27]	61	F	N.D.	F-A-R-LEU-S-L-HSM-EST-MAS	Pulse^‖^: twice, PSL (40), CyA (150), PE	TCZ/effective		PSL (40–6), CyA (100), MTX (4–6)	N.D.

8	Our patient	71	F	4 weeks	F-A-R-LEU-S-L-HSM-EST-MAS	Pulse^‖^: twice, PSL (60)	TCZ/effective		PSL (60)	PCP, CMV infection

AOSD, adult-onset Still's disease; MAS, macrophage activation syndrome; Pt, patient number; N.D., no data; F, fever; A, arthritis; R, skin rash; LEU, leukocytosis; S, sore throat; L, lymphadenopathies; HSM, hepatosplenomegaly; EST, elevated serum transaminases; Pulse^‖^, methylprednisolone pulse therapy; PSL, prednisolone; CyA, cyclosporine A; TAC, tacrolimus; MTX, methotrexate; PE, plasma exchange; IVIG, intravenous immunoglobulin; INF, infliximab; ANK, anakinra; TCZ, tocilizumab; ETN, etanercept; PCP, *Pneumocystis jirovecii* pneumonia; CMV, cytomegalovirus; ^a^used after first biologics; ^b^this patient continues to do well on TCZ as monotherapy after recovering from cardiac arrest.

**Table 2:** Case reports of biologic-associated macrophage activation syndrome after administration of biologics for the induction treatment of AOSD.

Pt	Reference	Age (years)	Sex	Disease duration of AOSD	Disease manifestations	Therapy before MAS developing (mg)	Biologics caused MAS	Therapy after biologic-associated MAS (mg)	Adverse events after biologics
1	Gianella et al. [17]	20	F	2 months	F-R-A-LEU-L-HSM	Ibuprofen	ETN	Pulse^‖^, PSL (100)	MAS, death

2	Kaneko et al. [18]	44	M	<7 months	F-A-R-LEU	PSL (50–30)	ETN	Pulse^‖^: twice, PSL (50), PE: twice, IVIG, AZA	MAS

3	Kobayashi et al. [19]	57	F	3 weeks	F-A-R-LEU-S-L-EST	PSL (80)	TCZ	DXP (0.2 mg/kg), CyA (2 mg/kg)	CMV infection, MAS, CDI

4	Banse et al. [20]	49	F	6 years	F-A-R-LEU-S-L	MTX (0.3 mg/kg), IFX, ANK, TCZ	Canakinumab	IVIG, PSL (1.5 mg/kg)	MAS

MAS, macrophage activation syndrome; AOSD, adult-onset Still's disease; Pt, patient number; F, fever; A, arthritis; R, skin rash; LEU, leukocytosis; S, sore throat; L, lymphadenopathies; HSM, hepatosplenomegaly; EST, elevated serum transaminases; DIC, disseminated intravascular coagulation; PSL, prednisolone; Pulse^‖^, methylprednisolone pulse therapy; MTX, methotrexate; IFX, infliximab; ANK, anakinra; TCZ, tocilizumab; CyA, cyclosporine A; PE, plasma exchange; DXP, dexamethasone palmitate; IVIG, intravenous immunoglobulin; AZA, azathioprine; CMV, cytomegalovirus; CDI, *Clostridium difficile* infection.
